# The association between hypoglycemia and glycemic variability in elderly patients with type 2 diabetes: a prospective observational study

**DOI:** 10.1186/s13098-021-00656-1

**Published:** 2021-04-01

**Authors:** Takahisa Handa, Akinobu Nakamura, Aika Miya, Hiroshi Nomoto, Hiraku Kameda, Kyu Yong Cho, So Nagai, Narihito Yoshioka, Hideaki Miyoshi, Tatsuya Atsumi

**Affiliations:** 1 Department of Rheumatology, Endocrinology and Nephrology, Faculty of Medicine and Graduate School of Medicine, Hokkaido University, N-15, W-7, Kita-ku, Sapporo, 060-8638 Japan; 2 Division of Diabetes and Endocrinology, Department of Medicine, NTT Sapporo Medical Center, Sapporo, Japan; 3 Division of Diabetes and Obesity, Faculty of Medicine and Graduate School of Medicine, Hokkaido University Graduate School of Medicine, Sapporo, Japan

**Keywords:** Coefficient of variation, Continuous glucose monitoring, Hypoglycemia

## Abstract

**Background:**

This study aimed to explore predictive factors of time below target glucose range (TBR) ≥ 1% among patients’ characteristics and glycemic variability (GV) indices using continuous glucose monitoring data in elderly patients with type 2 diabetes.

**Methods:**

We conducted a prospective observational study on 179 (71 female) Japanese outpatients with type 2 diabetes aged ≥ 65 years. The characteristics of the participants with TBR ≥ 1% were evaluated by multivariate logistic regression analysis. Receiver-operating characteristic (ROC) curve analyses of GV indices, comprising coefficient of variation (CV), standard deviation, and mean amplitude of glycemic excursions, were performed to identify the optimal index for the identification of patients with TBR ≥ 1%.

**Results:**

In the multivariate logistic regression analysis, none of the clinical characteristics, including HbA1c and C-peptide index, were independent markers for TBR ≥ 1%, while all three GV indices showed significant associations with TBR ≥ 1%. Among the three GV indices, CV showed the best performance based on the area under the curve in the ROC curve analyses.

**Conclusions:**

Among elderly patients with type 2 diabetes, CV reflected TBR ≥ 1% most appropriately among the GV indices examined.

*Trial registration* UMIN-CTR: UMIN000029993. Registered 16 November 2017

## Introduction

The number of elderly patients with diabetes increased to 111.2 million worldwide in 2019 [1], reflecting a substantial burden on the medical care system. The common features of these patients are the likelihood of having hypoglycemia associated with decreasing quality of life [2] and falls leading to bone fractures [3]. In addition, severe hypoglycemia was found to be associated with dementia [4], cardiovascular diseases and mortality risk [5] in these patients. Thus, elderly patients with diabetes require treatments that can prevent diabetic complications by stabilizing glycemic variability (GV) without hypoglycemia [6]. Consequently, it is important to identify hypoglycemia and clarify the characteristics of these patients with hypoglycemia using real-world data.

Recently, continuous glucose monitoring (CGM) has been used worldwide for daily clinical practice [7]. CGM traces the dynamical levels of glucose in interstitial fluid within subcutaneous fatty tissue over the whole day and night, making it possible to estimate GV and to detect hypoglycemia and hyperglycemia. An international consensus recommended the coefficient of variation (CV) as the main measure of GV, because it can predict hypoglycemia more accurately than other GV indices including the standard deviation (SD) [8]. Stable GV was defined as CV < 36% and unstable GV defined as CV ≥ 36%, reflecting the frequency of hypoglycemia in all age groups [9]. The international consensus also recommended percentage of time below target glucose range (TBR; < 70 mg/dL) < 1% as an appropriate CGM-related value for prevention of hypoglycemia in elderly patients with diabetes [10].

However, there are few clinical studies on the characteristics of elderly patients with diabetes and TBR ≥ 1% [11]. Furthermore, the relationships between TBR ≥ 1% and GV indices in CGM for elderly patients with diabetes remain to be fully characterized.

This study aimed to explore predictive factors of TBR ≥ 1% among patients’ characteristics and GV indices using continuous glucose monitoring data in elderly patients with type 2 diabetes.

## Materials and methods

### Study design and participants

The present study was a secondary analysis of a previous study with a multicenter (four sites), prospective observational design that utilized CGM [12]. Briefly, patients aged ≥ 20 years were included in this study when they consented to undergo ambulatory CGM without regard to levels of HbA1c, duration of diabetes, sex, and diabetic complications. The exclusion criteria were as follows: patients with type 1 diabetes; patients who had been hospitalized within the past 3 months; patients with diabetic ketosis or in diabetic coma, with serious infection, within preoperative or postoperative periods, or with trauma during the past 6 months; and patients administrated steroid treatments, were lactating or pregnant, or had difficulty consuming a normal diet. Patients provided clinical information (age, sex, anthropometric measurements, duration of diabetes in years, treatment regimen, and medical history), fasting blood samples, and CGM data. For the present study, we extracted data for patients aged ≥ 65 years among the enrolled patients.

The study was applied for the University Hospital Medical Information Network (UMIN) Center (registration number: UMIN 000029993). The study protocol was accepted by the Institutional Review Board at Hokkaido University Hospital Clinical Research and Medical Innovation Center (017-0147). It was conducted in accordance with the Declaration of Helsinki. Signed informed consent was obtained from all the patients.

### Biochemical analyses and data collection

Patients’ weight and height were measured using a calibrated scale. Body mass index (BMI) was calculated as weight in kilograms divided by height in meters squared. Further information including age, sex, treatment regimen, and medical history was taken with a questionnaire administered by the attending physicians. Blood samples were collected for this study after an overnight fast to measure the levels of HbA1c, fasting plasma glucose (FPG), C-peptide (CPR), and estimated glomerular filtration rate. The C-peptide index (CPI) was obtained by the following formula: 100 × fasting CPR (ng/mL)/plasma glucose (mg/dL), and applied to the reflection of endogenous insulin secretion [13].

Between 2018 and 2019, all patients went through ambulatory CGM for 14 consecutive days by means of the same technology (FreeStyle Libre Pro Sensor; Abbott Diabetes Care, Alameda, CA, USA). The CGM data remained blinded for patients and physicians because the CGM system used was a professional version for blinded CGM. We analyzed the CGM data for patients with at least 4 days of recorded data available. Data for the first and last days of wearing the device were excluded from the analysis because of concerns regarding the accuracy of the CGM system during attachment and detachment [14]. Several indices for GV were calculated using GlyCulator2 software [15], as follows: CV (100 × [SD of glucose]/[mean glucose]), SD, and mean amplitude of glycemic excursions (MAGE) [16]. Moreover, the target glucose range was set between 70 and 180 mg/dL in accordance with the international consensus recommendation [10]. We calculated three key CGM measurements for quality of glycemic control in clinical practice: percentage of readings and time per day in target glucose range (TIR; 70–180 mg/dL), TBR (< 70 mg/dL), and time above target glucose range (TAR; > 180 mg/dL) [10].

### Statistical analysis

As describes above, international consensus recommended TBR < 1% as an appropriate CGM-related value for prevention of hypoglycemia in elderly patients with diabetes [10]. Therefore, to determine the characteristics of elderly patients with diabetes who developed hypoglycemia, we assigned the patients to two subgroups: TBR ≥ 1% and TBR < 1%. For clinical factors related to TBR, comparisons between the two groups were performed by the Mann–Whitney U-test for means of continuous variables and Fisher’s exact test for proportions of categorical variables. Data are shown as mean ± standard deviation.

To analyze the characteristics of elderly patients with diabetes and TBR ≥ 1%, significant variables at value of *P* < 0.05 in univariate analysis were examined in multivariate logistic regression analysis for each GV index. To further determine the best GV index for identifying TBR ≥ 1% in elderly patients with diabetes as well as the optimal cut-off point for each GV index corresponding to TBR ≥ 1%, receiver-operating characteristic (ROC) curve analyses were carried out for the GV indices. All tests were two-sided, and *P* < 0.05 was taken to show statistical significance. All statistical analyses were carried out using JMP 14 software (SAS Inc., Cary, NC, USA).

## Results

**Fig. 1:**
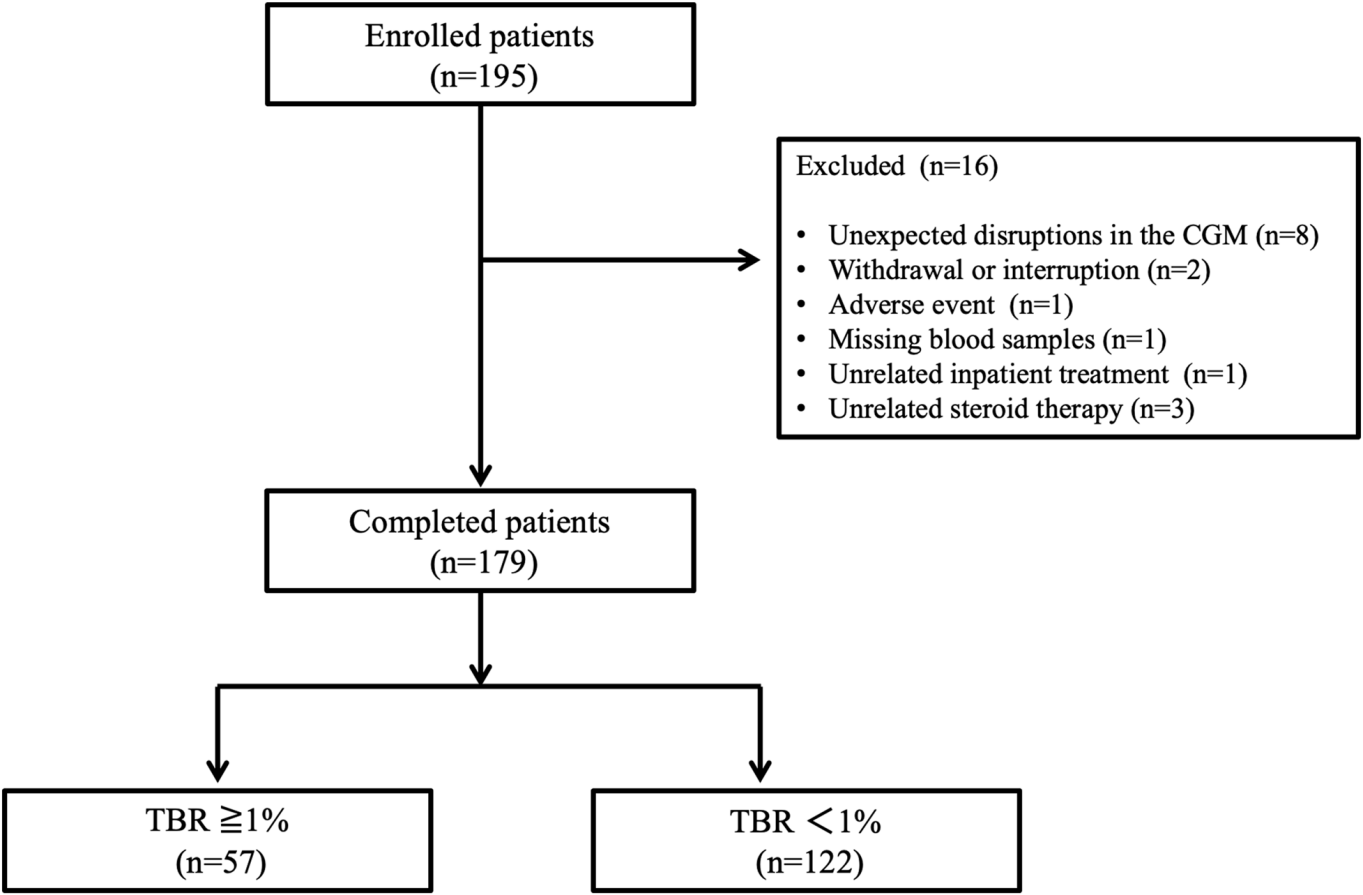
Flow chart of patients during the study

Among these 311 patients, 27 were excluded since they met at least one exclusion criteria described previously [12]. Furthermore, 105 patients aged < 65 years were excluded. The remaining 179 patients (71 females) were considered eligible and included in the analyses (Fig. 1).

The 179 elderly patients with type 2 diabetes were divided into two subgroups: TBR ≥ 1% (n = 57) and TBR < 1% (n = 122). Table 1 shows the anthropometric and biochemical characteristics of the full analysis group and the two subgroups. Compared with the TBR < 1% group, the TBR ≥ 1% group had significantly higher rate of insulin treatment and history of stroke or transient ischemic attack (TIA), and lower FPG, HbA1c, and CPI levels. For the three GV indices, CV, SD and MAGE were significantly higher in the TBR ≥ 1% group compared with the TBR < 1% group (Table 2). Mean monitored glucose in the TBR ≥ 1% group was significantly lower than that in the TBR < 1% group and TAR in the TBR ≥ 1% group was significantly lower than that in the TBR < 1% group although there was no significant difference in TIR between the two groups (Table 2).

**Table 1:** Comparisons of patient characteristics between the TBR ≥ 1% group and TBR < 1% group

	Total patients	TBR ≥ 1% group	TBR < 1% group	*P* value
*n*	179	57	122	
Age (years)	73.0 (68.0, 78.0)	74.0 (70.0, 79.0)	72.0 (68.0, 78.0)	0.0833
Females (%)	71 (39.7)	23 (40.4)	48 (39.3)	1.0000
BMI (kg/m^2^)	24.2 (22.0, 26.8)	23.6 (21.2, 25.1)	24.5 (22.1, 27.1)	0.0534
Diabetes duration (years)	16.0 (10.0, 24.0)	19.0 (12.5, 25.0)	15.0 (9.0, 24.0)	0.2620
Diabetic retinopathy	63 (35.4)	22 (38.6)	41 (33.9)	0.6148
Diabetic neuropathy	79 (49.4)	28 (51.9)	51 (48.1)	0.7386
Diabetic nephropathy	93 (52.0)	29 (50.9)	64 (52.7)	0.8735
Hypertension	134 (75.3)	43 (76.8)	91 (74.6)	0.8524
Dyslipidemia	124 (69.7)	41 (73.2)	83 (68.0)	0.5988
Hyperuricemia	32 (18.0)	10 (17.9)	22 (18.0)	1.0000
Diabetes treatment				
Any insulin (%)	74 (41.3)	36 (63.2)	38 (31.2)	< 0.0001
Sulfonylureas (%)	52 (29.1)	16 (28.1)	36 (29.5)	1.0000
Glinides (%)	27 (15.1)	11 (19.3)	16 (13.1)	0.3697
Metformin (%)	96 (53.6)	34 (59.7)	62 (50.8)	0.3346
Thiazolidine (%)	13 (7.3)	4 (7.0)	9 (7.4)	1.0000
α-Glucosidase inhibitors (%)	33 (18.4)	9 (15.8)	24 (19.7)	0.6796
DPP-4 inhibitors (%)	135 (75.4)	42 (73.7)	93 (76.2)	0.7131
GLP-1 receptor agonists (%)	18 (10.1)	6 (10.5)	12 (9.8)	1.0000
SGLT2 inhibitors (%)	30 (16.8)	6 (10.5)	24 (19.7)	0.1395
Acute coronary syndrome (%)	26 (14.5)	11 (19.3)	15 (12.3)	0.2560
History of stroke or TIA (%)	25 (14.0)	13 (22.8)	12 (9.8)	0.0350
FPG (mg/dL)	134.0 (116.0, 156.0)	119.0 (105.0, 135.5)	142 (123.0, 165.3)	< 0.0001
HbA1c (%)	7.1 (6.7, 7.7)	7.0 (6.6, 7.6)	7.2 (6.8, 7.8)	0.0387
HbA1c (mmol/mol)	54.1 (49.7, 60.7)	53.0 (48.6, 59.6)	55.2 (50.8, 61.7)	0.0387
CPI (ng/mL per mg/dL)	1.1 (0.7, 1.7)	1.0 (0.5, 1.5)	1.2 (0.9, 1.8)	0.0228
eGFR (mL/min/1.73 m^2^)	62.8 (48.4, 70.0)	58.0 (45.9, 68.3)	63.2 (50.6, 70.7)	0.1780

Values are expressed as median (interquartile range) or number (%) of patients in each category. The Mann–Whitney U-test or Fisher’s exact test was used for comparisons of the parameters between the TBR ≥ 1% group and TBR < 1% group

*BMI* body mass index, *DPP‐4* dipeptidyl peptidase‐4, *GLP‐1* glucagon‐like peptide‐1, *SGLT2* sodium–glucose cotransporter 2, *TIA* transient ischemic attack, *FPG* fasting plasma glucose, *CPI* C-peptide index, *eGFR* estimated glomerular filtration rate, *TBR* time below target glucose range

**Table 2:** Comparisons of GV indices and CGM measurements between two groups (TBR ≥ 1% and < 1%)

	Total patients	TBR ≥ 1% group	TBR < 1% group	*P* value
Mean monitored glucose (mg/dL)	145.5 (129.3, 163.2)	127.9 (117.0, 147.5)	149.0 (137.9, 168.7)	< 0.0001
CV (%)	27.9 (23.6, 33.8)	36.6 (30.0, 43.2)	26.0 (22.6, 29.0)	< 0.0001
SD (mg/dL)	40.1 (30.0, 51.4)	48.9 (35.5, 63.0)	38.6 (32.8, 48.0)	0.0012
MAGE	105.8 (86.5, 136.2)	128.2 (95.0, 158.5)	99.2 (83.5, 125.6)	0.0006
TIR (%)	77.4 (63.7, 87.0)	76.1 (63.0, 84.6)	78.1 (63.8, 87.2)	0.4058
TAR (%)	20.4 (11.4, 32.6)	15.8 (5.7, 27.4)	21.9 (12.4, 35.5)	0.0093
TBR (%)	0.2 (0.0, 2.1)	4.8 (2.2, 14.1)	0 (0.0, 0.2)	< 0.0001

Values are expressed as median (interquartile range) in each category. The Mann–Whitney U-test was used for comparisons of the parameters between the TBR ≥ 1% group and TBR < 1% group

*CGM* continuous glucose monitoring, *CV* coefficient of variation, *GV* glycemic variability, *SD* standard deviation, *MAGE* mean amplitude of glycemic excursions, *TIR* time in target glucose range, *TAR* time above target glucose range, *TBR* time below target glucose range

Multivariate logistic regression analysis showed that all three GV indices were independent predictive markers for TBR ≥ 1%. Specifically, CV (odds ratio [OR]: 1.43; 95% confidence interval [CI] 1.28–1.65), SD (OR: 1.27; 95% CI 1.17–1.41), and MAGE (OR: 1.07; 95% CI 1.04–1.10) were significantly associated with TBR ≥ 1% (Table 3). Although TAR was an independent predictive marker for TBR ≥ 1%, other clinical factors, including history of stroke or TIA, HbA1c, and CPI, were not independent markers. Mean monitored glucose was not included in the multivariate analysis because we found indications of multicollinearity between TAR and mean monitored glucose (data not shown). There was no difference in the proportion of patients who received insulin secretagogues (i.e., insulin and/or sulfonylurea or glinides) between the TBR ≥ 1% group and TBR < 1% group (data not shown). Although the TBR ≥ 1% group had a significantly lower rate of use of non-insulin secretagogues than the TBR < 1% group, CV, SD, and MAGE were identified as independent predictive markers for TBR ≥ 1% in multivariate logistic regression analysis even in patients using non-insulin secretagogues (data not shown). These results were similar to the findings in the whole study cohort.

**Table 3:** Clinical factors for TBR ≥ 1% analyzed by multivariate logistic regression analysis

	Odds ratio	95% CI	*P* value
CV			
Insulin treatment	1.49	0.54–4.13	0.4426
History of stroke or TIA	1.59	0.41–6.25	0.5056
HbA1c	1.17	0.45–3.13	0.7548
FPG	0.99	0.97–1.00	0.1420
CPI	1.09	0.67–1.92	0.7408
CV	1.43	1.28–1.65	< 0.0001
TAR	0.90	0.84–0.95	0.0005
SD			
Insulin treatment	1.76	0.68–4.54	0.2398
History of stroke or TIA	1.58	0.44–5.66	0.4856
HbA1c	0.99	0.42–2.33	0.9757
FPG	0.99	0.97–1.00	0.0956
CPI	1.03	0.65–1.71	0.8929
SD	1.27	1.17–1.41	< 0.0001
TAR	0.79	0.71–0.86	< 0.0001
MAGE			
Insulin treatment	2.35	0.93–5.94	0.0720
History of stroke or TIA	2.08	0.59–7.35	0.2535
HbA1c	1.13	0.51–2.53	0.7611
FPG	0.98	0.97–1.00	0.0850
CPI	0.90	0.57–1.45	0.6560
MAGE	1.07	1.04–1.10	< 0.0001
TAR	0.84	0.78–0.90	< 0.0001

*TIA* transient ischemic attack, *FPG* fasting plasma glucose, *CPI* C-peptide index, *CV* coefficient of variation, *SD* standard deviation, *MAGE* mean amplitude of glycemic excursions, *CI* confidence interval, *TAR* time above target glucose range, *TBR* time below target glucose range

**Fig. 2:**
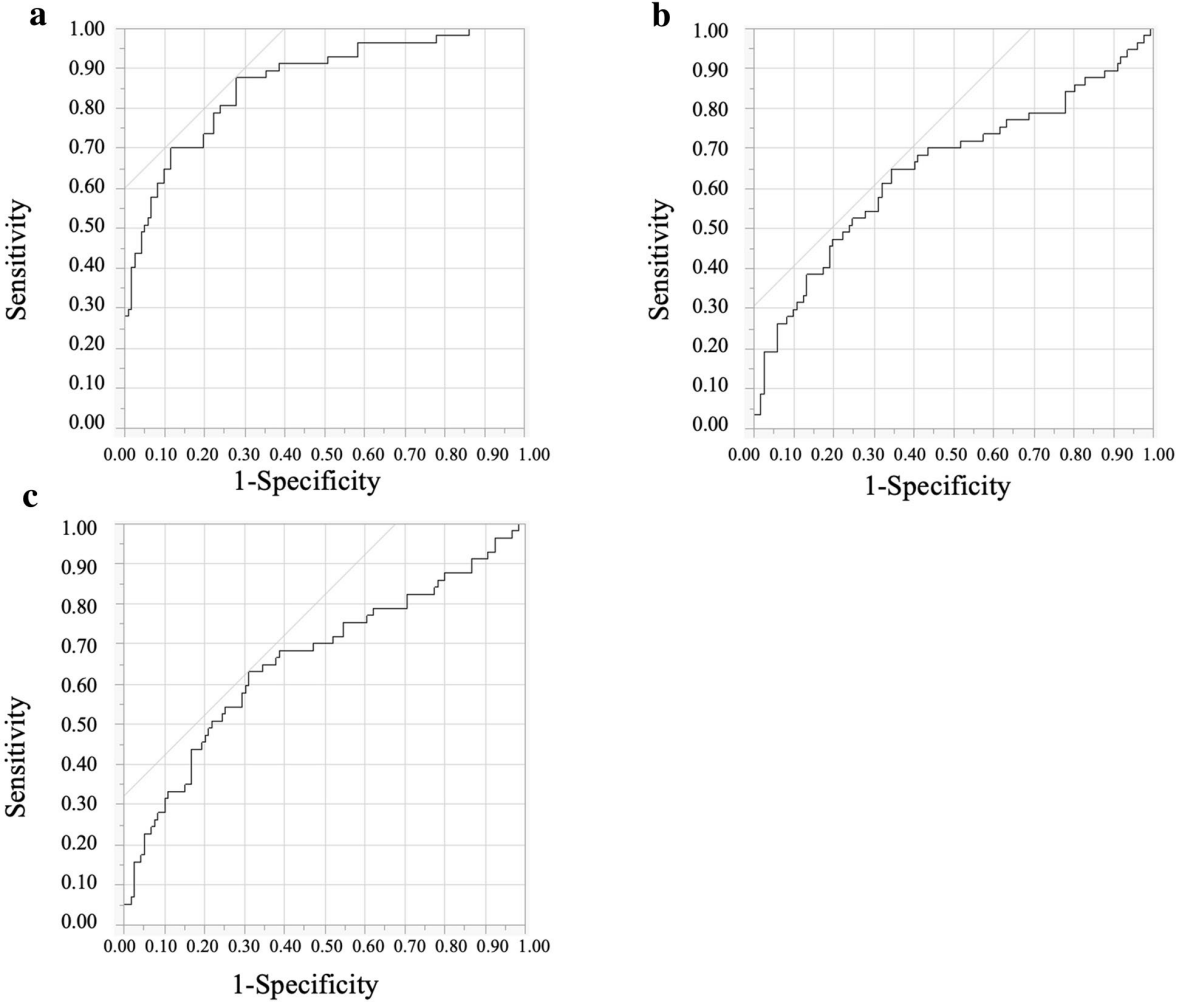
Comparison of AUCs for GV indices for predicting TBR ≥ 1% in receiver-operating characteristic curve analyses. The Figure shows the comparison of AUCs for glycemic variability indices for predicting TBR ≥ 1% in receiver-operating characteristic curve analyses for CV (**a**), SD (**b**), and MAGE (**c**). For CV, the cutoff point was 28.4 (AUC = 0.86; 95% CI 0.79–0.91). For SD, the cutoff point was 43.0 (AUC = 0.65; 95% CI 0.55–0.74). For MAGE, the cutoff point was 113.7 (AUC = 0.66; 95% CI 0.56–0.75). *AUC* area under the receiver-operating characteristic curve, *TBR* time below target glucose range, *CV* coefficient of variation, *SD* standard deviation, *MAGE* mean amplitude of glycemic excursions

Next, we constructed ROC curves, and calculated the areas under the ROC curve (AUCs) and the 95% CIs for all patients to assess the effects of CV, SD, and MAGE on TBR ≥ 1%. In the ROC curve analysis, CV had the best performance (AUC: 0.86; 95% CI 0.79–0.91; Fig. 2) and the optimal cut-off point for CV to predict TBR ≥ 1% was 28.4 (sensitivity: 87.7%; specificity; 72.1%). The optimal cut-off points for SD and MAGE were 43.0 (AUC: 0.65; 95% CI 0.55–0.74) and 113.7 (AUC: 0.66; 95% CI 0.56–0.75), respectively (Fig. 2).

## Discussion

In this study, we first demonstrated that the clinical characteristics of elderly patients with diabetes were not associated with TBR ≥ 1%. However, high GV indices contributed to TBR ≥ 1%. Among the three GV indices, CV was the most appropriate index for predicting TBR ≥ 1% in Japanese elderly outpatients with type 2 diabetes using CGM.

Regarding the relationships between clinical characteristics of patients with type 2 diabetes and hypoglycemia, a previous cross-sectional study showed that low level of fasting CPR was associated with high risk of hypoglycemia in 98 outpatients with type 2 diabetes using CGM [17]. While that study focused on patients of all ages who were all treated with insulin, our study only enrolled elderly patients aged ≥ 65 years (median age, 73.0 years), regardless of insulin therapy. For this reason, our results were different from ones in that study. Taken together, the findings suggest that the predictive markers for hypoglycemia in patients with diabetes differ between elderly patients and adult patients of all ages. Another cross-sectional study that enrolled elderly in-patients with type 2 diabetes who received insulin therapy showed that daytime and pre-midnight mean glucose levels were reliable markers to identify patients with an increased risk of nocturnal hypoglycemia [18]. In contrast, we enrolled outpatients regardless of insulin therapy. Taken together, the difference in patient background characteristics may have contributed to the inconsistent findings regarding hypoglycemia. Recently, an observation study involving CGM for 281 adult type 2 diabetes outpatients aged 40–75 years, including 181 patients aged 65–70 years, revealed that low HbA1c level and use of hypoglycemic agents related to hypoglycemia including sulfonylureas, glinides and insulin were associated with high TBR [11]. Although the subjects in that study were aged < 75 years, our study enrolled older patients (> 75 years) to provide more reflective data for real-world clinical practice. In addition, our study has the novelty of focusing on whether the clinical characteristics had associations with TBR ≥ 1% or < 1%, as the measure recommended by the international consensus to prevent hypoglycemia in elderly patients with diabetes. The result of our study showed that none of the clinical characteristics of these patients had correlations with TBR ≥ 1%. Elderly patients are at high risk of hypoglycemia related to drugs because of liver or renal dysfunction [19]. Furthermore, these patients are at high risk of severe hypoglycemia because of their characteristic of being likely to experience hypoglycemia unawareness [20]. Thus, it is desirable to identify the existence of hypoglycemia by making effective use of CGM regardless of any characteristics in elderly patients with diabetes.

Regarding the relationship between hypoglycemia and GV, a cross-sectional study on 294 Japanese inpatients with type 2 diabetes showed that CV and TBR were predictive markers for unstable GV [21]. These findings for enrolled inpatients of all ages may differ from those in elderly outpatients because less stringent targets for glycemic control are recommended in elderly patients [10]. The present study showed significant associations between TBR ≥ 1% and GV indices. Unstable GV was found to be associated with hypoglycemia in a previous observational study that performed self-monitoring of blood glucose in 335 adult patients (254 with type 1 diabetes and 81 with type 2 diabetes in all age groups) and computed the Average Daily Risk Range, one of the indices for GV [22]. Another observational study that performed CGM in 88 patients (20 with type 1 diabetes and 68 with type 2 diabetes in all age groups) showed that age had positive correlations with SD and MAGE in patients with type 2 diabetes [23]. These findings indicate that elderly patients with diabetes are at high risk of unstable GV. Although our study only focused on elderly patients with diabetes, a significant association between TBR ≥ 1% and GV was found. A retrospective study that enrolled elderly patients with type 2 diabetes showed that higher GV indicated a greater risk of hypoglycemia [24]. The study assessed elderly patients with poorly controlled diabetes whose mean HbA1c was 8.2%. In contrast, we assessed the same relationship in a cohort of patients whose median HbA1c was 7.1%. Taken together, unstable GV has an association with hypoglycemia in elderly patients with diabetes as well as in patients in all age groups.

Although CV was an indirect index that can predict hypoglycemia without being influenced by hyperglycemia, both SD and MAGE had a bias toward hyperglycemia due to the fact that these markers are absolute measures [25, 26]. A previous study showed that increasing TBR was associated with increases in CV [27]. In addition, CV was more strongly associated with a key CGM measure of hypoglycemia than other indices such as SD and MAGE in our previous prospective observational study in 284 Japanese patients with type 2 diabetes in all age groups [12]. In a prospective cohort study on patients with type 2 diabetes who had previous episodes of symptomatic hypoglycemia, CV was recommended as an index of glucose variability [28]. Similarly, the present study showed that CV was a more accurate predictor of TBR ≥ 1% than SD and MAGE, even in elderly patients with type 2 diabetes regardless of episode of hypoglycemia.

Our study showed that low TAR had a significant association with TBR ≥ 1%. While few studies have investigated the relationship between TAR and TBR in patients with type 2 diabetes, one previous study based on CGM data in 530 adults with type 1 diabetes or insulin-requiring type 2 diabetes showed that HbA1c had a positive relationship with TAR and an inverse relationship with TBR [29]. These findings were compatible with our results because low TAR could be correlated with high TBR.

There are some limitations in the present study. First, factors that can minimize GV remain to be determined because this study had a relatively small sample size. To identify such factors, a larger prospective longitudinal study is needed. Second, the validity of the CGM measurement quality could be a limitation. In this regard, these inaccuracies were minimized by excluding data from the first and last days of wearing the device [30].

## Conclusions

In conclusion, there were no associations between the clinical characteristics of Japanese elderly patients with type 2 diabetes and TBR ≥ 1% in a real-world clinical practice. Similar to previous studies on patients in all age groups, CV had the most significant association with hypoglycemia among the GV indices examined. In elderly patients with diabetes, it is preferable to perform evaluations using CGM to confirm good blood glucose profiles without hypoglycemia.

## Data Availability

The datasets used and/or analyzed during the current study are available from the corresponding author on reasonable request.

## Abbreviations

AUC: The areas under the ROC curve
BMI: Body mass index
CGM: Continuous glucose monitoring
CI: Confidence interval
CKD: Chronic kidney disease
CPI: C-peptide index
CV: Coefficient of variation
DPP-4: Dipeptidyl peptidase-4
eGFR: Estimated glomerular filtration rate
FPG: Fasting plasma glucose
GLP-1: Glucagon like peptide-1
GV: Glycemic variability
HbA1c: Glycated hemoglobin
MAGE: Mean amplitude of glycemic excursions
QOL: Quality of life
ROC: Receiver operating characteristic
SD: Standard deviation
SGLT2: Sodium glucose cotransporter 2
TAR: Time above target glucose range
TBR: Time below target glucose range
TIA: Transient ischemic attack
TIR: Time in target glucose range

## References

[1] Pouya S, Inga P, Paraskevi S (2019). Global and regional diabetes prevalence estimates for 2019 and projections for 2030 and 2045: results from the International Diabetes Federation Diabetes Atlas, 9th edition. Diabetes Res Clin Pract.

[2] Laiteerapong N, Karter AJ, Liu JY (2011). Correlates of quality of life in older adults with diabetes: the diabetes & aging study. Diabetes Care.

[3] Johnston SS, Conner C, Aagren M (2012). Association between hypoglycaemic events and fall-related fractures in medicare-covered patients with type 2 diabetes. Diabetes Obes Metab.

[4] Yaffe K, Falvey CM, Hamilton N (2013). Association between hypoglycemia and dementia in a biracial cohort of older adults with diabetes mellitus. JAMA Intern Med.

[5] Zoungas S, Patel A, Chalmers J (2010). Severe hypoglycemia and risks of vascular events and death. N Engl J Med.

[6] Rosenzweig JL, Conlin PR, Gonzalvo JD (2020). 2019 endocrine society measures set for older adults with type 2 diabetes who are at risk for hypoglycemia. J Clin Endocrinol Metab.

[7] American Diabetes Association (2021). 7. Diabetes technology: standards of medical care in diabetes-2021. Diabetes Care.

[8] Danne T, Nimri R, Battelino T (2017). International consensus on use of continuous glucose monitoring. Diabetes Care.

[9] Monnier L, Colette C, Wojtusciszyn A (2017). Toward defining the threshold between low and high glucose variability in diabetes. Diabetes Care.

[10] Battelino T, Danne T, Bergenstal RM (2019). Clinical targets for continuous glucose monitoring data interpretation: recommendations from the international consensus on time in range. Diabetes Care.

[11] Kuroda N, Kusunoki Y, Osugi K (2020). Relationships between time in range, glycemic variability including hypoglycemia and types of diabetes therapy in Japanese patients with type 2 diabetes mellitus: Hyogo diabetes hypoglycemia cognition complications study. J Diabetes Investig.

[12] Miya A, Nakamura A, Handa T (2020). Impaired insulin secretion predicting unstable glycemic variability and time-below-range in type 2 diabetes regardless of HbA1c or diabetes treatment. J Diabetes Investig.

[13] Funakoshi S, Fujimoto S, Hamasaki A (2011). Utility of indices using C-peptide levels for indication of insulin therapy to achieve good glycemic control in Japanese patients with type 2 diabetes. J Diabetes Investig.

[14] Bailey T, Bode BW, Christiansen MP (2015). The performance and usability of a factory-calibrated flash glucose monitoring system. Diabetes Technol Ther.

[15] Pagacz K, Stawiski K, Szadkowska A (2018). GlyCulator2: an update on a web application for calculation of glycemic variability indices. Acta Diabetol.

[16] Service FJ, Molnar GD, Rosevear JW (1970). Mean amplitude of glycemic excursions, a measure of diabetic instability. Diabetes.

[17] Merete MB, Gæde P, Hommel E (2020). Glycaemic variability and hypoglycaemia are associated with C-peptide levels in insulin-treated type 2 diabetes. Diabetes Metab.

[18] Klimontov VV, Myakina NE (2017). Glucose variability indices predict the episodes of nocturnal hypoglycemia in elderly type 2 diabetic patients treated with insulin. Diabetes Metab Syndr.

[19] Moen MF, Zhan M, Hsu VD (2009). Frequency of hypoglycemia and its significance in chronic kidney disease. Clin J Am Soc Nephrol.

[20] Bremer JP, Jauch-Chara K, Hallschmid M (2009). Hypoglycemia unawareness in older compared with middle-aged patients with type 2 diabetes. Diabetes Care.

[21] Torimoto K, Okada Y, Hajime M (2018). Risk factors of hypoglycemia in patients with type 2 diabetes mellitus: a study based on continuous glucose monitoring. Diabetes Technol Ther.

[22] Kovatchev BP, Otto E, Cox D (2006). Evaluation of a new measure of blood glucose variability in diabetes. Diabetes Care.

[23] Tanaka C, Saisho Y, Tanaka K (2014). Factors associated with glycemic variability in Japanese patients with diabetes. Diabetol Int.

[24] Ishikawa T, Koshizaka M, Maezawa Y (2018). Continuous glucose monitoring reveals hypoglycemia risk in elderly patients with type 2 diabetes mellitus. J Diabetes Investig.

[25] Kovatchev BP (2017). Metrics for glycaemic control—from HbA1c to continuous glucose monitoring. Nat Rev Endocrinol.

[26] Jin SM, Kim TH, Bae JC (2014). Clinical factors associated with absolute and relative measures of glycemic variability determined by continuous glucose monitoring: an analysis of 480 subjects. Diabetes Res Clin Pract.

[27] Rodbard D (2020). Glucose time in range, time above range, and time below range depend on mean or median glucose or HbA1c, glucose coefficient of variation, and shape of the glucose distribution. Diabetes Technol Ther.

[28] Gómez AM, Muñoz OM, Marin A (2018). Different indexes of glycemic variability as identifiers of patients with risk of hypoglycemia in type 2 diabetes mellitus. J Diabetes Sci Technol.

[29] Hirsch IB, Welsh JB, Calhoun P (2019). Associations between HbA1c and continuous glucose monitoring-derived glycaemic variables. Diabet Med.

[30] Tsoukas M (2020). Accuracy of FreeStyle Libre in adults with type 1 diabetes: the effect of sensor age. Diabetes Technol Ther.

